# Kaposi’s Sarcoma Associated Herpesvirus Encoded Viral FLICE Inhibitory Protein K13 Activates NF-κB Pathway Independent of TRAF6, TAK1 and LUBAC

**DOI:** 10.1371/journal.pone.0036601

**Published:** 2012-05-08

**Authors:** Hittu Matta, Ramakrishnan Gopalakrishnan, Ciaren Graham, Bhairavi Tolani, Akshat Khanna, Han Yi, Yulan Suo, Preet M. Chaudhary

**Affiliations:** Jane Anne Nohl Division of Hematology and Center for the Study of Blood Diseases, University of Southern California Keck School of Medicine, Los Angeles, California, United States of America; Lisbon University, Portugal

## Abstract

**Background:**

Kaposi’s sarcoma associated herpesvirus encoded viral FLICE inhibitory protein (vFLIP) K13 activates the NF-κB pathway by binding to the NEMO/IKKγ subunit of the IκB kinase (IKK) complex. However, it has remained enigmatic how K13-NEMO interaction results in the activation of the IKK complex. Recent studies have implicated TRAF6, TAK1 and linear ubiquitin chains assembled by a linear ubiquitin chain assembly complex (LUBAC) consisting of HOIL-1, HOIP and SHARPIN in IKK activation by proinflammatory cytokines.

**Methodology/Principal Findings:**

Here we demonstrate that K13-induced NF-κB DNA binding and transcriptional activities are not impaired in cells derived from mice with targeted disruption of *TRAF6*, *TAK1* and *HOIL-1* genes and in cells derived from mice with chronic proliferative dermatitis (cpdm), which have mutation in the *Sharpin* gene *(Sharpin^cpdm/cpdm^)*. Furthermore, reconstitution of NEMO-deficient murine embryonic fibroblast cells with NEMO mutants that are incapable of binding to linear ubiquitin chains supported K13-induced NF-κB activity. K13-induced NF-κB activity was not blocked by CYLD, a deubiquitylating enzyme that can cleave linear and Lys63-linked ubiquitin chains. On the other hand, NEMO was required for interaction of K13 with IKK1/IKKα and IKK2/IKKβ, which resulted in their activation by “T Loop” phosphorylation.

**Conclusions/Significance:**

Our results demonstrate that K13 activates the NF-κB pathway by binding to NEMO which results in the recruitment of IKK1/IKKα and IKK2/IKKβ and their subsequent activation by phosphorylation. Thus, K13 activates NF-κB via a mechanism distinct from that utilized by inflammatory cytokines. These results have important implications for the development of therapeutic agents targeting K13-induced NF-κB for the treatment of KSHV-associated malignancies.

## Introduction

Transcription factors of the nuclear factor-κ B (NF-κB) family regulate expression of hundreds of genes involved in the inflammatory and immune response [1], [2], [3]. The classical NF-κB complex is a heterodimer of the p65/RelA and p50 subunits and is found in most cells in association with a family of inhibitory proteins, called IκBs [4], [5]. The IκB proteins are identified by the presence of multiple ankyrin repeats and retain NF-κB in the cytoplasm through the masking of the nuclear localization signal of p65. Most of the diverse signaling pathways that activate NF-κB converge on a multi-subunit IκB kinase (IKK) complex that contains two catalytic subunits, IKK1/IKKα and IKK2/IKKβ, and a regulatory subunit, NEMO/IKKγ [5], [6]. The IKK complex induces phosphorylation of IκB proteins that leads to their ubiquitination and proteasomal-mediated degradation, allowing the NF-κB subunits to enter the nucleus and turn on the expression of their target genes [4].

A kinase complex composed of TAK1 and its associated subunits TAB1, TAB2 and TAB3 has been reported to act as the upstream kinase that activates the IKK complex by targeting two serine residues located in the conserved “T loop” region of the kinase domains of IKK1/IKKα and IKK2/IKKβ [7], [8]. Recently, various members of the E3 ligase family have recently been implicated in NF-κB activation by participating in ubiquitination processes not linked to protein degradation [8]. For example, one of the best characterized pathways that results in NF-κB activation is the Tumor Necrosis Factor Receptor 1 (TNFR1) signaling pathway, which is activated by TNFα [9], [10]. Binding of TNFα to TNFR1, results in the recruitment of several adaptor proteins to the cytosolic domain of the receptor, including the adaptor protein TRADD, E3 ligase TRAF2 and cIAPs and kinases RIP, TAK1 and IKKs [10], [11], [12], [13], [14], [15], [16]. These macromolecular complex results in TRAF2 and cIAP1-mediated Lys63-linked polyubiquitination of RIP, which then recruits the TAK1 complex through specific affinity of TAB2 and TAB3 for Lys63-linked chains [7], [8], [17]. Recently, another novel ubiquitin ligase complex, designated LUBAC (linear ubiquitin chain assembly complex), was also shown to be part of the TNFR1 signaling complex and to play a key role in NF-κB activation by catalyzing the assembly of linear ubiquitin chains [18], [19], [20], [21].

Activation of NF-κB by IL1β and lipopolysaccharide involve similar components. Binding of these ligands to their receptors initially results in the recruitment of MyD88 [22]. Subsequently, MyD88 recruits kinases IRAK4 and IRAK1 and E3 ligase TRAF6, which results in IRAK1 phosphorylation, release of IRAK1/TRAF6 and TRAF6 autoubiquitination [23], [24], [25]. Ubiquitinated TRAF6 is then believed to recruit the TAK1 complex and in concert with Ubc13, modify both TAK1 and IRAK1 with Lys63-linked chains [26], [27]. The final step in this process involves recruitment of the IKKs through NEMO and their phosphorylation/activation by TAK1 [26], [28].

Kaposi’s sarcoma (KS) associated herpesvirus (KSHV), also known as Human herpesvirus 8 (HHV8), is the commonest cause of malignancy among patients with Acquired Immunodeficiency Syndrome [29], [30]. Infection with KSHV has been associated with KS, primary effusion lymphoma (PEL) and multicentric Castleman’s disease (MCD) [31]. KSHV encodes a vFLIP (viral FLICE inhibitory Protein), encoded by the open reading frame K13, which is one of the few viral proteins to be expressed in cells latently-infected with KSHV [32]. Based on its structural homology to the prodomain of FLICE/caspase 8 [33], K13 was initially believed to be an inhibitor of FLICE/caspase 8 (hence the name vFLIP) [33]. However, subsequent work by our laboratory and others have demonstrated that the main biological function of K13 is the activation of the NF-κB pathway rather than inhibition of caspase 8/FLICE, and that it utilizes this pathway to promote cellular survival, proliferation, transformation and cytokine secretion [34], [35], [36], [37], [38]. We recently demonstrated that K13 bypasses TRAF2 and TRAF3 and activates the NF-κB pathway by directly binding to the NEMO subunit of the IKK complex [39]. However, it is not clear how K13-NEMO interaction results in the activation of the IKK complex. In this study, we have investigated the role of TRAF6, TAK1 and LUBAC in K13-induced NF-κB activation. We demonstrate that K13 activates the NF-κB pathway independent of TRAF6 and TAK1. Furthermore, linear polyubiquitination of NEMO is not required for K13-induced NF-κB activation. Instead, NEMO is required for mediating the interaction between K13 and IKK1 and IKK2. Our results suggest that K13 activates the NF-κB pathway using a mechanism distinct from that utilized by proinflammatory cytokines.

## Materials and Methods

### Cell Lines and Reagents

293T, BC1, BCBL1, Jurkat and Namalwa cells were obtained from ATCC (Manassas, VA). BCBL1 and Namalwa cells expressing an empty vector and K13 have been described previously [39], [40]. Wild-type and NEMO-deficient cells have also been described previously [41]. *TRAF6* ^+/+^ and *TRAF6* ^−/−^ mouse embryonic fibroblasts (MEFs) were gifts from Tak Mak (University of Toronto, Canada) [24]. *TAK1* ^+/+^ and *TAK1* ^−/−^ MEFs were generously provided by Dr. Stephanie S. Watowich (MD Anderson Cancer Center, Houston, Texas). *HOIL-1* ^−/−^ and *cpdm* cells were kindly provided by Dr. Kazuhiro Iwai (Osaka University, Japan). TAK1 inhibitor 5Z-7-oxo-zeaenol was purchased from Tocris Bioscience (Ellisville, MO), recombinant TNFα and IL-1β were from Peprotech (Rocky Hill, NJ), and 4-Hydroxytamoxifen (4OHT) was purchased from Sigma (St. Lois, MO).

### Plasmids

Plasmids encoding K13 and 4-Hydroxytamoxifen (4OHT)-inducible K13-ER^TAM^, CYLD, EDAR (ectodermal dysplasia receptor) and NEMO have been described previously [36], [37], [39], [42], [43]. Retroviral constructs expressing NEMO mutants defective in linear ubiquitination were kindly provided by Dr. Ivan Dikic (Goethe University Medical School). Recombinant retroviruses were generated and used to generate polyclonal populations of stably transduced MEFs following selection with puromycin essentially as described previously [44].

### Luciferase Reporter Assay

 293T cells were transfected in a 24-well plate with various test plasmids along with an NF-κB luciferase reporter construct (75 ng/well) and a pRSV/LacZ (β-galactosidase) reporter construct (75 ng/well) as described previously [42]. Cells were lysed 24–36 h later, and extracts were used for the measurement of firefly luciferase and β-galactosidase activities, respectively. Luciferase activity was normalized relative to the β-galactosidase activity to control for the difference in the transfection efficiency. Transient transfection of MEFs and measurement of luciferase activity was performed essentially as described previously [45]. Briefly, MEFs were transfected in duplicate using Lipofectamine 2000 (Invitrogen, Carlsbad, CA) in a 24-well plate with the various test plasmids along with an NF-κB/luciferase reporter construct (75 ng/well) and a *Renilla* luciferase reporter construct (phRG-TK, 75 ng/well, Promega, Madison, WI). The cells were lysed 48 hours later, and extracts were used for the measurement of firefly and *Renilla* luciferase activities as described in the Dual-Luciferase® Reporter (DLR™) Assay system from Promega. Firefly luciferase activity was normalized relative to the *Renilla* luciferase activity to control for the difference in the transfection efficiency.

### Western Blot

Western blot analysis was performed essentially as described previously [34]. Primary antibodies used in these experiments were: NEMO, Total-IκBα, Rel B, TRAF6 (Santa Cruz Biotechnology, Santa Cruz, CA); tubulin, M2 FLAG (Sigma, St. Louis, MO), and phospho-TAK1, phospho-IκBα and A20 (Cell Signaling, Danvers, MA). A mouse monoclonal antibody against K13 (8F6) was raised in our laboratory.

### NF-κB DNA-binding Assays

The NF-κB subunit composition of the K13-induced NF-κB complexes in wild-type and *TAK1^−/−^* MEFs was determined with an NF-κB ELISA kit (TransAM NF-κB; Active Motif, Carlsbad, CA) according to the manufacturers’ instructions. The electrophoretic mobility shift assay was performed as described previously [34].

### Pathscan ELISA Assay

The PathScan Phospho-IKKα (Ser176/180), Phospho-IKKβ (Ser177/181) sandwich ELISA Kits and PathScan Phospho-IκBα (Ser32) sandwich ELISA antibody pair (Cell Signaling, Danvers, MA) were used to detect endogenous levels of IKKα, IKKβ and IκBα proteins when phosphorylated at Ser176/180, Ser177/181 and Ser32, respectively.

### Statistical Analyses

Two-tailed paired Student’s *t* test was used to test for differences between two groups. Differences with a p≤0.05 were considered as statistically significant. All experiments were repeated a minimum of three times with duplicate/triplicate samples.

## Results

### TRAF6 is not Required for K13-induced NF-κB Activation

**Figure 1:**
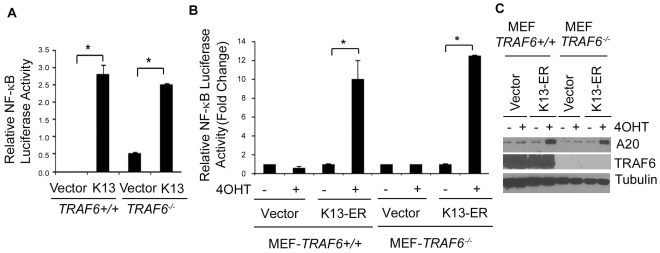
TRAF6 is not required for K13-induced NF-κB activation. **A.** *TRAF6^+/+^* and *TRAF6^−/−^* MEFs were transfected with a control vector or vector encoding K13 along with an NF-κB-Luc construct (75 ng/well) and a Renilla reporter construct (75 ng/well, normalization control) using Lipofectamine 2000-mediated transaction. The luciferase reporter assay was performed 48 hours post transfection essentially as described in “Materials and Methods” section. Asterisks (*) indicate significance at levels of p≤0.05. **B.** *TRAF6^+/+^* and *TRAF6^−/−^* MEFs were transfected with a control vector or a vector encoding K13-ER^TAM^ along with an NF-κB-Luc construct and a Renilla reporter construct. The transfected cells were subsequently treated with 4OHT (20 nM) for 48 hours and the reporter assays performed as described above. The values shown are averages (mean±SE) of one representative experiment out of three in which each transfection was performed in duplicate. Asterisks (*) indicate significance at levels of p≤0.05. **C.** *TRAF6^+/+^* and *TRAF6^−/−^* MEF cells expressing an empty vector or the FLAG-tagged K13-ER^TAM^ treated with 4OHT were examined by immunoblot analysis for upregulation of A20. Tubulin was used as a loading control.

Different members of the TRAF family are required for NF-κB activation by distinct stimuli. Thus, while TRAF2 is known to be required for NF-κB activation by TNFα, TRAF6 has been implicated in the activation of this pathway signaling via interleukin 1 and Toll like receptors [46], [47]. We have recently demonstrated that TRAF2 is not involved in K13-induced NF-κB activation [39]. To rule out the involvement of TRAF6 in K13-induced NF-κB activity, we transiently transfected *TRAF6^+/+^* and *TRAF6^−/−^* MEFs with an empty vector or a K13 expression construct and examined the activation of a cotransfected NF-κB-Luc reporter construct. As shown in Figure 1A, we observed near equivalent K13-induced NF-κB-Luc activity in the *TRAF6^+/+^* and *TRAF6^−/−^* MEFs. Essentially similar results were obtained when the experiment was repeated using the K13-ER^TAM^ construct followed by treatment with 4OHT (Fig. 1B). Finally, we generated stable populations of *TRAF6^+/+^* and *TRAF6^−/−^* MEFs expressing an empty vector or the K13-ER^TAM^ construct. The mutated estrogen receptor (ER^TAM^) in the K13-ER^TAM^ construct does not bind to its physiological ligand estrogen but binds with very high affinity to the synthetic ligand 4OHT (4-hydroxytamoxifen) and allows the control of K13 activity in a 4OHT-dependent fashion [48]. We treated the resulted cells with 4OHT to activate K13 activity and assessed the activation of the NF-κB pathway by measuring the upregulation of A20, a protein known to be induced by K13-induced NF-κB activity [49]. As shown in Fig. 1C, treatment with 4OHT resulted in equivalent upregulation of A20 in the *TRAF6^+/+^* and *TRAF6^−/−^,* which argues against the involvement of TRAF6 in K13-induced NF-κB activation.

### TAK1 is not Required for K13-induced NF-κB Activation

**Figure 2:**
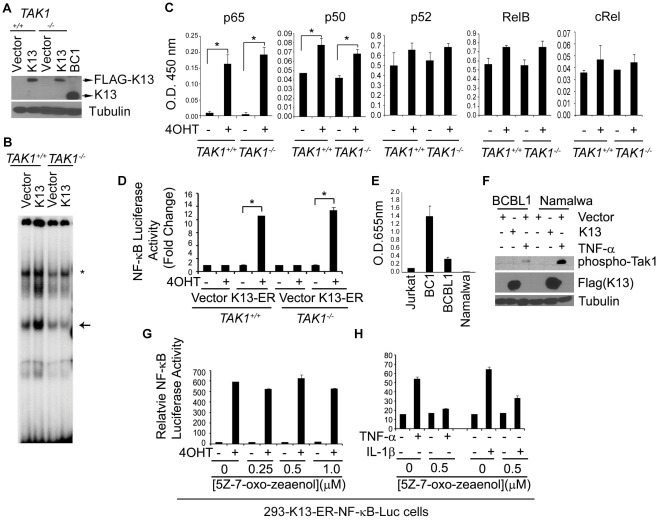
K13 activates NF-κB pathway without involving TAK1. A. Immunoblot showing expression of ectopically expressed FLAG-tagged K13 in *TAK1^+/+^* and *TAK1^−/−^* MEFs and endogenous K13 in BC1 cell line. **B.** Status of NF-κB pathway, as measured by an EMSA in *TAK1^+/+^* and *TAK1^−/−^* MEF cells stably expressing vector and K13. The position of the induced NF-κB complexes is marked by an *asterisk*, while an *arrow* marks the position of the constitutive complexes. **C.** The NF-κB subunits composition of the K13-induced nuclear NF-κB complexes in the wild-type and *TAK1^−/−^* MEFs was determined with an ELISA-based DNA-binding assay performed in triplicate. Asterisks (*) indicate significance at levels of p≤0.05. **D.** *TAK1^+/+^* and *TAK1^−/−^* MEFs expressing FLAG-K13-ER^TAM^ were transfected with NF-κB-Luc and Renilla reporter constructs and subsequently treated with 4OHT (20 nM) for 48 hours and the reporter assays performed as described in Figure 1A. Asterisks (*) indicate significance at levels of p≤0.05. **E.** Nuclear extracts from Jurkat, BC1, BCBL1 and Namalwa were used for the measurement of p65 DNA-binding activity using an ELISA-based assay. **F.** Lack of phosphorylation of TAK1 by K13. Cell lysates prepared from BCBL1 and Namalwa cells expressing an empty vector or FLAG-K13 were probed with an antibody to detect phosphorylation of TAK1. Vector cells treated with TNFα were used a positive control. The blot was re-probed with FLAG and Tubulin antibodies to check the expression of the transduced K13 and equal protein loading, respectively. **G**−**H.** Treatment with TAK1 inhibitor 5Z-7-oxo-zeaenol had no effect on 4OHT induced NF-κB-Luc activity in 293-K13-ER^TAM^-NF-κB-Luc cells but effectively blocked TNFα and IL1β induced NF-κB-Luc activity.

TRAF6 is believed to activate NF-κB by activating TAK1, which in turn phosphorylates and activates the IKK complex resulting in NF-κB activation [7], [8]. TAK1 was also shown to be required for NF-κB activation by KSHV-encoded viral G protein coupled receptor (vGPCR) [50]. To study the role of TAK1 in K13-induced NF-κB activation, we took advantage of murine embryonic fibroblast (MEFs) cells deficient in this protein. We used retroviral-mediated gene transfer to generate stable pools of wild-type and *TAK1*-deficient MEFs expressing an empty vector and FLAG-tagged K13. An immunoblot analysis confirmed equivalent expression of the K13 in the wild-type (WT) and *TAK1^−/−^* MEFs (Fig. 2A). The expression of K13 in the wild-type (WT) and *TAK1^−/−^* MEFs, however, was lower than its expression in the KSHV-infected BC1 cell line (Fig. 2A). We subsequently used an electrophoretic mobility shift assay (EMSA) to compare the ability of K13 to activate the NF-κB pathway in the two cell lines. Consistent with the known ability of K13 to activate the NF-κB pathway, ectopic expression of K13 in the *TAK^+/+^* MEFs resulted in increased nuclear NF-κB DNA binding activity (Fig. 2B). However, an equivalent increase in NF-κB DNA binding activity was observed upon ectopic expression of K13 in the *TAK1^−/−^* cells (Fig. 2B). To confirm these results, we generated stable clones of WT and *TAK1^−/−^* MEFs expressing the K13-ER^TAM^ fusion protein and used an ELISA-based DNA-binding assay to study the subunit composition of NF-κB complexes induced following treatment with 4OHT in the two cell lines. This assay revealed that p65 and p50 were the major NF-κB subunits induced by 4OHT in both the WT and *TAK1* ^−/−^ K13-ER^TAM^ expressing MEFs, with modest induction of p52, RelB and cRel subunits (Fig. 2C). Thus, TAK1 has no major effect on either the amount or subunit composition of NF-κB complexes induced by K13. We next examined the effect of TAK1 on K13-induced NF-κB transcriptional activity. For this purpose, we transiently transfected an empty vector or a K13-ER^TAM^-fusion construct in wild-type and *TAK1^−/−^* MEFs and measured NF-κB transcriptional activation using an NF-κB luciferase reporter assay. As shown in Figure 2D, transfection of *TAK^+/+^* MEFs with K13-ER^TAM^ followed by treatment with 4OHT resulted in a significant increase in NF-κB-Luc reporter activity. However, 4OHT treatment of K13-ER^TAM^-transfected *TAK^−/−^* MEFs also led to an equivalent increase in NF-κB-Luc activity, thereby demonstrating that TAK1 is not required for K13-induced NF-κB transcriptional activation.

### K13 does not Induce TAK1 Phosphorylation

Human T-cell Leukemia virus 1 (HTLV-1) encoded Tax protein resembles K13 in activating the NF-κB pathway by binding to NEMO [51]. It was recently shown that TAK1 is constitutively activated by Thr-187 phosphorylation in Tax-positive HTLV-1-transformed T cells [52]. Therefore, to examine whether K13 would similarly induce TAK1 Thr-187 phosphorylation, we used retroviral-mediated gene transfer and generated polyclonal populations of two lymphoma cell lines, BCBL1 and Namalwa, expressing an empty vector and FLAG-K13. We chose these cell lines as they possess low to none constitutive NF-κB activity endogenously (Fig. 2E). Ectopic expression of K13 in both BCBL1 and Namalwa cells failed to induce phosphorylation of TAK1 as determined by Western blotting with a phospho-Thr184/Thr187 TAK1 antibody, whereas treatment with TNFα successfully did so (Fig. 2F). Thus, in contrast to HTLV-1 oncoprotein Tax, K13 does not induce TAK1 phosphorylation.

### K13-induced NF-κB is not Blocked by a Specific Inhibitor of TAK1

5Z-7-Oxozeaenol, a resorcyclic lactone of fungal origin, is a potent and selective inhibitor of TAK1 [53]. To confirm the lack of involvement of TAK1 in K13-induced NF-κB activity, we studied the effect of increasing doses of 5Z-7-Oxozeaenol treatment on 4OHT induced NF-κB luciferase activity in 293NF-κB-Luc- K13-ER^TAM^ cells which stably express an NF-κB-driven luciferase reporter construct and the K13-ER^TAM^ fusion protein. As shown in Figure 2G, treatment with up to 1 µM 5Z-7-Oxozeaenol had no significant inhibitory effect on 4OHT-induced NF-κB reporter activity. In comparison, 0.5 µM 5Z-7-Oxozeaenol significantly inhibited TNFα and IL-1β-induced NF-κB activity in the 293NF-κB-Luc reporter cells (Fig. 2H). Taken collectively with previous studies, the above results confirm that TAK1 is not required for K13-induced NF-κB activation.

### HOIL-1 is not Essential for K13-induced NF-κB Activation

**Figure 3:**
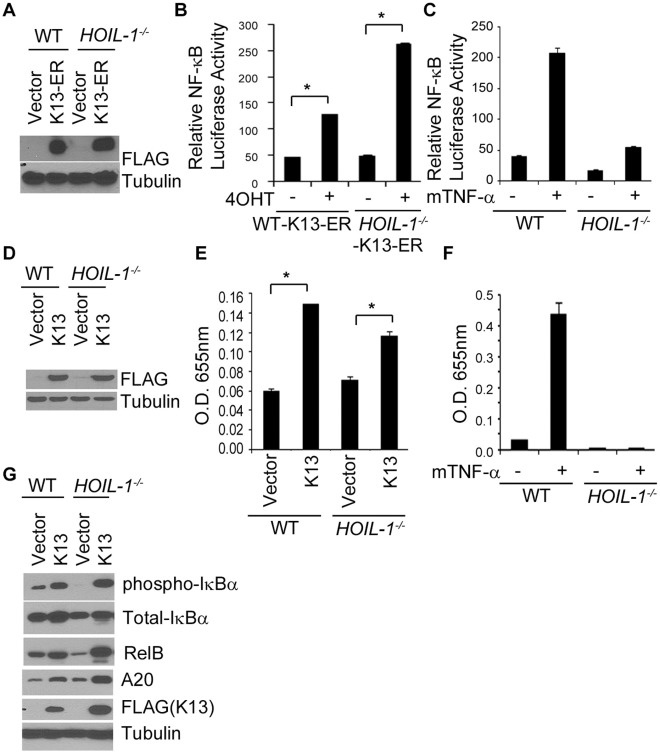
HOIL-1 is not essential for K13-induced NF-κB activation. **A.** The expression of FLAG-tagged K13-ER^TAM^ in wild-type and *HOIL-1^−/−^* MEF was confirmed with Western blotting. The blot was re-probed with a tubulin antibody to show equal protein loading. **B.** Wild-type and *HOIL-1^−/−^* MEFs stably expressing FLAG-K13-ER^TAM^ were transfected with NF-κB-Luc and Renilla reporter constructs. Cells were subsequently treated with 4OHT (20 nM) for 48 hours and the luciferase reporter assay was performed essentially as described in Figure 1A. Asterisks (*) indicate significance at levels of p≤0.05 as compared to vehicle-treated cells. **C.** Wild-type and *HOIL-1^−/−^* MEFs were transfected with NF-κB-Luc and Renilla reporter constructs and 6 hours post-transfection, these cells were treated with mTNF-α (10ng/ml) for 18 hours and the luciferase reporter assay was performed essentially as described in Figure 1A. **D.** Expression of transduced FLAG-tagged K13 in wild-type and *HOIL-1^−/−^* MEFs was examined by immunoblot analysis; tubulin was used as a loading control. **E.** Nuclear p65 DNA binding activities in the nuclear extracts of wild-type and *HOIL-1^−/−^* MEFs expressing an empty vector or FLAG-K13. Asterisks (*) indicate significance at levels of p≤0.05 as compared to vector cells. **F.** Nuclear p65 DNA binding activities in the nuclear extracts of wild-type and *HOIL-1^−/−^* MEFs following treatment with murine TNFα. **G.** Wild-type and *HOIL-1^−/−^* MEFs expressing FLAG-K13 were examined for NF-κB activation by Western blot analysis using antibodies against phospho-IκBα, Total IκBα, A20 and RelB. The blot was re-probed with FLAG and Tubulin antibodies to check the expression of the transduced K13 and equal protein loading, respectively.

Recently, a novel ubiquitin ligase complex, designated LUBAC (linear ubiquitin chain assembly complex), which is composed of two RING finger proteins, HOIL-1 (also known as RBCK1) and HOIP, and SHARPIN (SHANK-associated RH domain interacting protein in postsynaptic density) was shown to play a key role in the activation of the NF-κB pathway by binding to NEMO and conjugating linear polyubiquitin chains onto its specific Lys residues [18], [19], [20]. We used *HOIL-1*-null MEFs to test the involvement of LUBAC-induced linear polyubiquitination in K13-induced NF-κB activation. For this purpose, we generated stable populations of wild-type and *HOIL-1*-null MEFs expressing and empty vector and FLAG-tagged K13-ER^TAM^ construct. A Western blot analysis confirmed expression of the K13-ER^TAM^-fusion protein in both wild-type and *HOIL-1^−/−^* MEFs, with slightly higher expression in the latter (Fig. 3A). We measured NF-κB transcriptional activation in the resulting cells with and without 4OHT treatment using an NF-κB luciferase reporter assay. As shown in Figure 3B, we observed robust activation of NF-κB-Luc activity upon 4OHT treatment in both the WT-K13-ER^TAM^ and *HOIL-1* ^−/−^ K13-ER^TAM^ cells, arguing against the possibility that HOIL-1 is required for K13-induced NF-κB activity. Indeed, the *HOIL-1^−/−^* K13-ER^TAM^ cells showed a greater increase in NF-κB-Luc activity as compared to the WT-K13-ER^TAM^ cells upon 4OHT treatment, which was probably due to the higher level expression of the K13-ER^TAM^ protein in the former. Consistent with the lack of requirement of HOIL-1 in K13-induced NF-κB activity, we observed equivalent increase in nuclear p65 DNA-binding activity in the wild-type and *HOIL-1* ^−/−^ MEFs upon ectopic K13 expression (Fig. 3D–E). In contrast, TNFα-induced NF-κB-Luc and p65 DNA binding activities were markedly reduced in *HOIL-1* ^−/−^ MEFs (Fig. 3C, 3F). Finally, to confirm the lack of involvement of *HOIL-1* in K13-induced NF-κB activity, we used Western blot analysis to compare the phosphorylation of IκBα and expression of A20 and RelB, two proteins that are known to be upregulated by the NF-κB pathway, in the wild-type and *HOIL-1* ^−/−^ MEFs stably expressing K13. As shown in Figure 3G, we observed increased phosphorylation of IκBα and upregulation of A20 and RelB in both wild-type and *HOIL-1^−/−^* MEFs upon ectopic K13 expression, which argues against an essential role of HOIL-1 in K13-induced NF-κB activity. Indeed, K13-expressing *HOIL-1^−/−^* MEFs showed slightly more IκBα phosphorylation and A20 and RelB expression as compared to the K13-expressing wild-type MEFs. This was probably due to slightly higher level of ectopic K13 expression in the latter (Fig. 3G).

### Sharpin is not Essential for K13-induced NF-κB Activation

**Figure 4:**
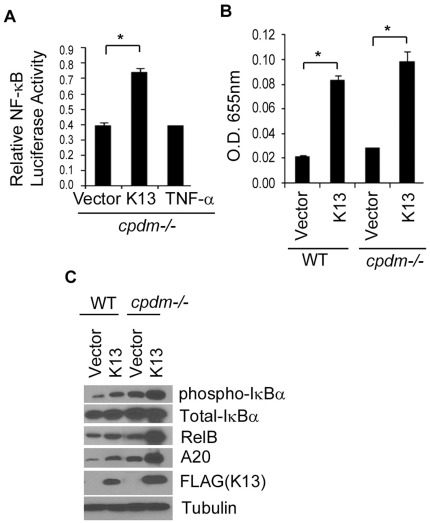
SHARPIN is not required for K13-induced NF-κB activation. **A.** MEFs deficient in SHARPIN (cpdm) were able to activate NF-κB reporter activity in the presence of K13, but not with TNFα. Asterisks (*) indicate significance at levels of p≤0.05. **B.** SHARPIN is not required for K13-induced NF-κB p65 DNA binding as determined by ELISA. Asterisks (*) indicate significance at levels of p≤0.05 as compared to vehicle-treated cells. **C.** Total cell lysates from wild-type, and *cpdm* MEF stably expressing an empty vector or FLAG-tagged K13 were used to examine the requirement of SHARPIN on K13-induced NF-κB activation by upregulation of expression of A20, RelB, phosphorylation of IκBα and degradation of total IκBα. The expression of the FLAG-tagged K13 protein and equal protein loading was confirmed by blotting with FLAG and Tubulin antibodies, respectively.

Mutation in *Sharpin* (SHANK-associated RH domain interacting protein in postsynaptic density) gene are responsible for *cpdm* (chronic proliferative dermatitis) phenotype in mice that resembles the phenotype of patients with X-linked hyper-IgM syndrome and hypohydrotic ectodermal dysplasia (XHMED), which is caused by mutations in NEMO [54]. SHARPIN shows significant similarity to HOIL-1 and was recently shown to be a component of LUBAC and to be required for linear ubiquitination of NEMO and NF-κB activation [19], [20], [21]. To examine whether SHARPIN is required for K13-induced NF-κB activity, we examined the ability of K13 to activate an NF-κB-Luc reporter construct in *cpdm* MEFs. As shown in Figure 4A, transient transfection of K13 strongly activated the NF-κB-Luc reporter in *cpdm* MEFs, whereas treatment with TNFα failed to do so. We also generated stable populations of wild-type and *cpdm* MEFs expressing K13 and observed equivalent increase in the nuclear p65/RelA DNA-binding activity upon ectopic K13 expression (Fig. 4B). Finally, ectopic K13 expression also resulted in an increase in IκBα phosphorylation and enhanced expression of the NF-κB target proteins A20 and RelB in the *cpdm* MEFs (Fig. 4C). Taken collectively, the above results demonstrate that SHARPIN is not essential for K13-induced NF-κB activity.

### NEMO Mutants Defective in Binding to Linear Polyubiquitin Chains Support K13-Induced NF-κB

**Figure 5:**
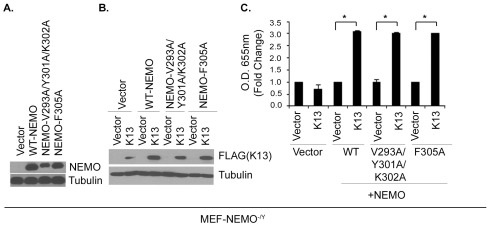
The ability of K13 to activate NF-κB pathway does not require linear polyubiquitination of NEMO. A–B. MEF NEMO^−/Y^ cells were stably transduced with wild-type NEMO and two NEMO mutants defective in linear ubiquitination of NEMO (NEMO-Y293A/Y301A/K302A and NEMO-F305A) and their expression was confirmed by immunoblotting using NEMO antibody. These cells were further transduced with either an empty retroviral vector or a retroviral vector expressing FLAG-tagged K13 and their expression was determined by immunoblotting with FLAG antibody. The blot was re-probed with a tubulin antibody to show equal loading of protein. **C.** Upon expression of K13, these cells were able to activate NF-κB p65 DNA binding as determined by ELISA. The values shown are averages (mean±SE) of one representative experiment out of three in which the level of p65 DNA binding was measured in triplicate. Asterisks (*) indicate significance at levels of p≤0.05.

NEMO was recently shown to selectively bind linear ubiquitin chains and NEMO mutants V293A/Y301A/K302A and F305A that are defective in binding linear ubiquitin chains were shown to be incapable of supporting NF-κB activation by TNFα and other agonists [55]. Therefore, as an independent test of the involvement of linear polyubiquitination in K13-induced NF-κB activity, we reconstituted NEMO-deficient MEFs with an empty vector, wild-type NEMO, NEMO-V293A/Y301A/K302A and NEMO-F305A using retroviral mediated gene transfer (Fig. 5A). In the resulting cells, we stably expressed an empty vector and K13 (Fig. 5B). After confirming the equivalent expression of the transduced proteins by Western blotting, we examined the status of the NF-κB by measuring nuclear p65/RelA DNA binding activity. Ectopic expression of K13 failed to induce nuclear p65/RelA DNA binding activity in the *NEMO^−/Y^* MEFs reconstituted with an empty vector, but resulted in approximately 3 fold increase in the nuclear p65/RelA DNA binding activity in those reconstituted with the wild-type NEMO, thereby confirming that NEMO is required for K13-induced NF-κB activation (Fig. 5C). More importantly, we observed an equivalent increase in K13-induced nuclear p65/RelA DNA binding activity in *NEMO^−/Y^* MEFs reconstituted with NEMO-V293A/Y301A/K302A and NEMO-F305A mutants, respectively (Fig. 5C). Thus, binding of NEMO to linear polyubiquitin chain is not required for K13-induced NF-κB activation.

### CYLD Fails to Block K13-induced NF-κB Activity

**Figure 6:**
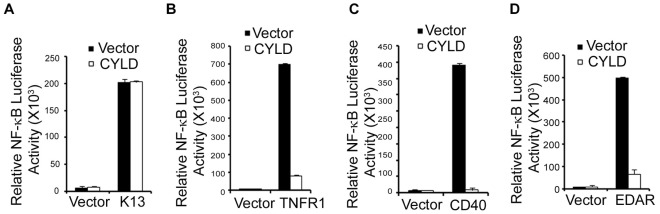
Deubiquitinating enzyme CYLD, a negative regulator of NF-κB pathway does not block K13-induced NF-κB activity. A–D. The 293T cells were co-transfected with a control vector or vectors encoding FLAG-K13, TNFR1, CD40 and EDAR (250 ng/ml) and a CYLD plasmid construct (750 ng/well) along with NF-κB-Luc reporter construct (75 ng/well) and a pRSV/LacZ (β-galactosidase) reporter construct (75 ng/well), and the reporter assay performed as described in “Materials and Methods” The values shown are averages (mean±SE) of one representative experiment out of three in which each transfection was performed in duplicate.

The deubiquitylating enzyme CYLD, a negative regulator of the NF-κB pathway, can cleave linear ubiquitin chains [56], [57]. As another independent test to rule out the involvement of linear Ub chains in K13-induced NF-κB activation, we tested the ability of CYLD to block K13-induced NF-κB-Luc reporter activity. We used TNFR1, CD40, and EDAR (Ectodermal Dysplasia Receptor) as controls for this experiment since NF-κB activation by these receptors is known to require linear polyubiquitination or is inhibited by CYLD [58], [59]. As shown in Figure 6A-D, coexpression of CYLD had no effect on K13-induced NF-κB activity but effectively blocked TNFR1, CD40 and EDAR-induced NF-κB activity. Thus, in contrast to the receptors of the TNFR family, K13-induced NF-κB is not dependent on linear ubiquitin chains.

### Essential Role of NEMO in Mediating K13-IKK Interaction and IKK Activation

**Figure 7:**
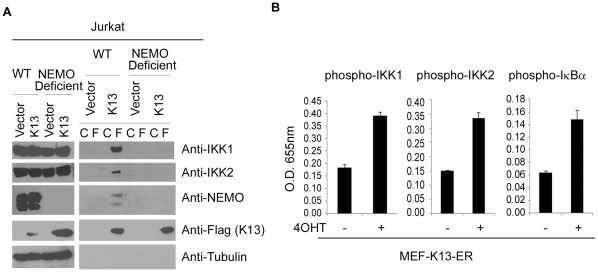
NEMO-mediated recruitment of IKK1 and IKK2 to K13 and their activation by “T loop” phosphorylation. **A.** Co-immunoprecipitation assay showing NEMO is essential for mediating the interaction of K13 with IKK1 and IKK2. FLAG-tagged K13 was immunoprecipitated from WT and NEMO-deficient Jurkat cells using control (C) and FLAG (F) antibody beads and presence of co-immunoprecipitated IKK1, IKK2 and NEMO detected by immunoblotting. **B.** MEFs expressing K13-ER^TAM^ were treated with 4OHT and phosphorylation status of IKK1, IKK2 and IκBα was determined using Pathscan phospho-IKK1 (Ser176/180), phospho-IKK2 (Ser177/181), phosphor-IκBα (Ser32) ELISA kits, respectively.

We and others have previously demonstrated that K13 interacts with NEMO and NEMO is essential for K13-induced NF-κB activation [34], [60]. The studies in the preceding section clearly demonstrate that TAK1, TRAF6 and linear polyubiquitination of NEMO are not involved in K13-induced NF-κB activation. Therefore, to explain the role of NEMO in K13-inducd NF-κB activation, we tested the hypothesis that NEMO mediates the interaction between K13 and IKK1 and IKK2. For this purpose, FLAG-tagged K13 was immunoprecipitated from the WT and NEMO-deficient Jurkat cells using Flag or control antibody beads and its interaction with endogenously expressed NEMO, IKK1 and IKK2 was examined by Western blot analysis. As shown in Figure 7A, while interaction of K13 with NEMO, IKK1, and IKK2 was readily detected in samples immunoprecipitated with Flag antibody from wild-type Jurkat cells, no significant interaction between K13 and IKK1 or IKK2 was observed in the NEMO-deficient Jurkat cells, Stimulation of cells with TNFα or IL-1 induces recruitment of IKK1 and IKK2 to NEMO and their subsequent phosphorylation and activation [61], [62]. The relevant phosphorylation sites in IKK1 and IKK2 have been mapped to regions in their catalytic domain that shares strong homology with “T loop” regulatory sequences found in the members of the mitogen activated protein kinase kinase (MAP2K) family of enzymes [61], [62]. These sites include Ser176 and Ser180 for IKK1 and Ser177 and Ser181 for IKK2. We next examined if recruitment of IKK1 and IKK2 to the K13-NEMO complex is also associated with “T Loop” phosphorylation of IKK1, IKK2 or both. The K13-ER^TAM^ expressing MEFs were treated with 4OHT and phosphorylation status of IKK1 and IKK2 was determined using Pathscan phospho-IKK1(Ser176/180) and phospho-IKK2 (Ser177/181) ELISA kits, respectively. As shown in Figure 7C, 4OHT treatment of K13-ER^TAM^ cells resulted in a significant increase in “T loop” phosphorylation of IKK1, IKK2 as compared to the untreated cells. The K13-induced increased “T loop” phosphorylation of IKK1 and IKK2 was also associated with increased phosphorylation of IκBα. Thus, recruitment of IKK1 and IKK2 to the K13-NEMO complex results in their activation by “T loop” phosphorylation.

## Discussion

K13 is known to activate the NF-κB pathway by binding to activating the ∼700 kDa IKK complex consisting of IKK1/IKKα, IKK2/IKKβ and NEMO/IKKγ [34]. We have previously shown that NEMO is essential for K13-induced NF-κB activation [34], [45]. However, the exact mechanism by which K13-NEMO interaction results in the activation of the IKK complex and the NF-κB pathway is not clear. In a recent study, we demonstrated that TRAF2, which is required for NF-κB activation by TNFα, is not required for the interaction of K13 with NEMO and for K13-induced NF-κB activation [39]. In this report, we have excluded the involvement of TRAF6, which has been implicated in NF-κB activation during signaling emanating from IL-1 and Toll like receptors [46], [47], in K13-induced NF-κB activation. Taken collectively, these studies support the model that K13 bypasses TRAFs and directly interacts with the IKK complex to activate the NF-κB pathway. This model is consistent with previous studies demonstrating a direct interaction between K13 and NEMO/IKKγ in GST-pull down and yeast two-hybrid assays [18], and the recently described crystal structure of the K13-NEMO complex [63].

We next asked the question how the interaction of K13 with NEMO results in the activation of the IKK complex and the NF-κB pathway. Genetic and pharmacological studies have implicated TAK1 as the upstream kinase responsible for the activation of the IKK complex during NF-κB activation by pro-inflammatory cytokines [7], [8]. A recent study also showed that TAK1 is required for NF-κB activation by KSHV-encoded viral G protein coupled receptor (vGPCR) [50]. In contrast, we found no defect in K13-induced NF-κB DNA-binding and transcriptional activities in *TAK1*-deficient cells. The lack of involvement of TAK1 in K13-induced NF-κB activity was further supported by our studies with 5Z-7-oxo-zeaenol, a specific inhibitor of TAK1, which failed to block K13-induced NF-κB transcriptional activity.

Recent studies have implicated LUBAC-mediated linear polyubiquitination in NF-κB activation by proinflammatory cytokines [18], [19], [20]. Depending on their position relative to the substrate, the ubiquitin monomers in uniquitin chains can be described as Ub_distal_ or Ub_proximal_ subunits [55]. A crystal structure of NEMO in complex with linear ubiquitin molecules has demonstrated that distinct residues of NEMO interact with Ub_distal_ and Ub_proximal_ subunits [55]. A recent study reported that reconstitution of a NEMO-deficient clone of mouse 70Z/3 lymphoma cells, designated 1.3E2, with a murine NEMO mutant that is defective in binding to Ub_distal_ (NEMO-F312A) resulted in restoration of K13-induced NF-κB activation [64]. To confirm the lack of involvement of linear polyubiquitination in K13-induced NF-κB activation, we took advantage of cells that are deficient in two essential components of LUBAC, an enzyme complex required for linear polyubiquitination. Our results with *HOIL-1^−^/^−^* and *cpdm* MEFs, rule out the requirement of LUBAC-induced linear polyubiquitination in K13-induced NF-κB activation. The lack of requirement of linear polyubiquitination in K13-induced NF-κB activation was further supported by our results showing that CYLD, a deubiquitylating enzyme that can cleave linear ubiquitin chains, cannot block K13-induced NF-κB activity. Taken collectively, the above results demonstrate that linear polyubiquitination is not required for K13-induced activation of the IKK complex and the NF-κB pathway.

In addition to its interaction with linear ubiquitin chains, NEMO has been shown to interact with Lys63-linked ubiquitin chains [8], [65] and it has been recently suggested that both types of chains cooperate for optimal activation of the NF-κB pathway by proinflammatory cytokines [66]. Interestingly, a F312A mutation in human NEMO, which corresponds to the F305A mutation in mouse NEMO tested in this study, was shown to be defective in binding to Lys63-linked ubiqutin chains [8], which suggests that K13 activates NF-κB independent of Lys63-linked chains as well. This hypothesis is further supported by our results showing that K13-induced NF-κB is not blocked by CYLD, a deubiquitylating enzyme that cleaves both linear and Lys63-linked ubiquitin chains [57], [67].

Based on the lack of involvement of TRAF6, TAK1 and LUBAC in K13-induced NF-κB activation, we support a model according to which the interaction between K13 and NEMO is direct and is not mediated via the TRAF family members or the linear ubiquitin chains. We further demonstrate that binding of K13 to NEMO is required for recruitment of IKK1 and IKK2 to K13. Taken collectively, the above results support the model according to which binding of K13 to NEMO manipulates the latter into an open conformation that facilitates the recruitment of IKK1 and IKK2 and their subsequent activation by “T loop” phosphorylation.

Specific inhibitors of IKK (particularly IKK2) that can selectivity block NF-κB pathway are being developed by several pharmaceutical companies [68]. However, since the NF-κB pathway plays a key role in normal immune response [69], the clinical utility of such agents in KSHV-associated lymphoproliferative disorders is likely to be limited by their potential toxicity. For example, *in vivo* administration of ML120B, a small molecule selective inhibitor of IKK2, led to rapid depletion of T and B cells [70]. Although no IKK1-selective inhibitor has been described to date, genetic studies suggest that IKK1 may similarly play an essential role in lymphocyte development and function [71]. As patients with KSHV-associated malignancies are usually immunosuppressed, the deleterious effect of IKK inhibitors on lymphocyte number and function is of potential concern. Therefore, we believe that rather than blocking NF-κB pathway globally, the ideal agent for the treatment of KSHV-associated lymphoproliferative disorders should selectively block K13-induced NF-κB activation. Our results showing that K13 activates the NF-κB pathway via a mechanism distinct from that utilized by immune and inflammatory cytokines suggest that it may be possible to design such an agent that will selectively block K13-induced NF-κB while sparing the physiological activation of this pathway required for normal lymphocyte development, survival and function.
